# Impacts of heavy metals on early development, growth and reproduction of fish – A review

**DOI:** 10.1016/j.toxrep.2022.04.013

**Published:** 2022-04-20

**Authors:** Khanam Taslima, Md Al-Emran, Mohammad Shadiqur Rahman, Jabed Hasan, Zannatul Ferdous, Md Fazle Rohani, Md Shahjahan

**Affiliations:** a Department of Fisheries Biology and Genetics, Bangladesh Agricultural University, Mymensingh 2202, Bangladesh; b Laboratory of Fish Ecophysiology, Department of Fisheries Management, Bangladesh Agricultural University, Mymensingh 2202, Bangladesh; c Bangamata Sheikh Fojilatunnesa Mujib Science and Technology University, Melandah, Jamalpur, Bangladesh; d Department of Aquaculture, Bangladesh Agricultural University, Mymensingh 2202, Bangladesh

**Keywords:** Aquaculture, Embryos, Larvae, Pollution, Trace elements

## Abstract

Heavy metals pollution causes a threat to the aquatic environment and to its inhabitants when their concentrations exceed safe limits. Heavy metals cause toxicity in fish due to their non-biodegradable properties and their long persistence in the environment. This review investigated the effects of heavy metals on early development, growth and reproduction of fish. Fish embryos/larvae and each developmental stage of embryo respond differently to the intoxication and vary from species to species, types of metals and their mode of actions, concentration of heavy metals and their exposure time. Many of the heavy metals are considered as essential nutrient elements that positively improve the growth and feed utilization of fishes but upon crossing the maximum tolerable limit these metals cause not only a hazard to fish health but also to human consumers and the disruption of ecological systems. Reduced gonadosomatic index (GSI), fecundity, hatching rate, fertilization success, abnormal shape of reproductive organs, and finally failure of reproduction in fish have been attributed to heavy metal toxicity. In summary, this review sheds light on the manipulation of fish physiology by heavy metals and seeks to raise sensitivity to the prevention and control of aquatic environmental contamination, particularly from heavy metals.

## Highlights

• Heavy metals in water exceed the safe limits, causes toxicity in fish is a global concern.
• Describes the occurrences of heavy metals in water.
• Provides a general view on the effects of heavy metals on fish.

## Graphical Abstract

**Figure:**
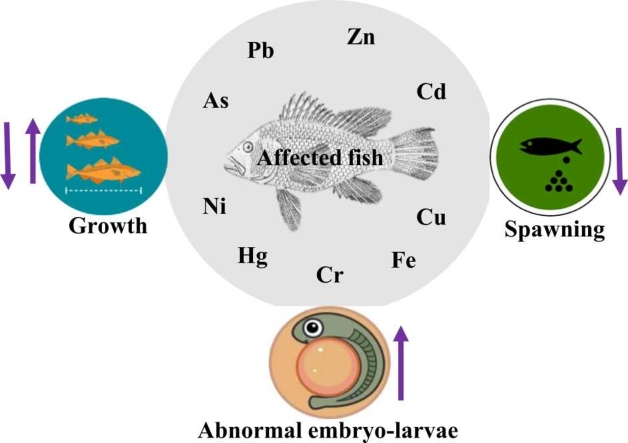

## 1 Introduction

Heavy metals pollution is a great concern to aquatic environments because they impart a wide range of toxicities with serious impacts to the aquatic faunal communities [1], [2]. Most of the heavy metals accumulated in aquatic water bodies are originate from anthropogenic activities such as agricultural cultivation, erosions of landfills, docking and embarking activities, sewage from industrial and domestic wastewater and some natural processes [1], [3]. The uncontrolled population growth, intensive agricultural activities and heavy industrialization result in a wide range of pollutants which eventually inflict serious consequences on aquatic ecosystems as well as associated faunal and floral communities [4], [5], [6]. Commonly, trace amount of heavy metals (non-degradable) cause serious difficulties in aquatic systems as a result of their assimilation, deposition and even incorporation at a specific concentration in abiotic substances and ultimately, accumulated into the body of associated aquatic organisms [7]. Heavy metals accumulate into the tissues of aquatic organisms throughout different aquatic food chains where they can be concentrated; bioaccumulated metals can result in substantial human health hazards upon consumption of these contaminated aquatic foods [8]. The rapid growth of industrialization across the cities results in the release of effluents contaminated with toxic metals including chromium (Cr), nickel (Ni), copper (Cu), lead (Pb), iron (Fe), and zinc (Zn). In broad, metals can be classified as biologically essential and nonessential. Metals like aluminum (Al), cadmium (Cd), mercury (Hg), tin (Sn) and lead (Pb) have no records of specific biological functions and therefore their toxicities rise with high concentration. On the other hand, essential metals (Cr, Zn, Ni, Cu, Co, Fe) have established biological functions and toxicities occur in response to either their deficiencies or excessive concentrations. Essential metals positively improved the growth and feed utilization of several species [9], [10], [11], [12], [13], [14], [15] but when maximum allowable/tolerable limit these metals are exceeded, they hamper the normal physiological and ecological systems in the aquatic environment [16], [17], causing toxicity within the organisms and ultimately causing a substantial threat to human health [1], [8]. Most of these heavy metals are highly carcinogenic in nature and in addition they can cause serious health complexities like liver disorders, cardiovascular difficulties, kidney dysfunctions and in extreme cases death. Heavy metal pollution severely disrupts the physiology of several aquatic organisms, especially fish [4], [18], [19]. Heavy metal contamination greatly changed the hemato-biochemical scenario of fish and also resulted several deformities (cellular and nuclear) in different blood cells [19], [20], [21]. Genetic damages as a result of heavy metal toxicities have also been recorded by several studies [18]. Heavy metals contamination significantly hampers the reproductive performances of fish [22], [23], [24]. Investigations have reported several reproductive compromises including reduced GSI, fecundity, hatching rate, fertilization success, abnormal shape of reproductive organs, and finally overall reproductive success in response to a variety of heavy metals [25], [26], [27], [28], [29], [30]. Moreover, heavy metals severely affected the embryonic and larval development of fish through resulting number of complexities such as increased heart rate, reduced cardiac activity, increased mortality rate, deformed shape, vertebral column deformities etc. in different developmental stages of embryo [11], [31], [32], [33], [34], [35]. Despite the destructive impacts of several heavy metals on fish physiology and reproductive performance in fishes, few if any generalized or comprehensive patterns of these responses are available. The current review focuses on the aggregation of up-to-date information about the impacts of heavy metals on embryonic and larval development, growth, reproductive performance with an emphasis of the most commercially important aquaculture species.

## 2 Heavy metals effects on embryonic and larval development of fish

Early developmental stages of fish, specifically embryos and larvae, are more susceptible to pollutants such as heavy metals than juvenile and adult fish are, and are widely used as bio-indicators to determine the toxicity of such chemicals to the aquatic organisms [36], [37]. Various endpoints such as developmental malformations (teratogenicity), physiological and biochemical alterations, behavioural and functional deformities are used to assess and predict the toxicity of heavy metals to fish population [35]. Fish embryos/larvae at each developmental stage of embryo (blastula, gastrula, segmentation, hatching etc.) respond differently to the intoxication and vary from species to species, types of metals and their mode of actions, concentration of heavy metals and their exposure time etc. [38], [39]. For instance, hatching and embryo survival of African catfish (*Clarias gariepinus*) were unaffected by Cd exposure at a concentration ranging from 0.05-5 mg/L. Another study reported that embryo and larvae survival, hatching of Ide (*Leuciscus idus)* were significantly affected by Cd exposure (100 μg/L; [35], [40]. The types of deformities in different fish species due to expose to different heavy metals are summarized in Table 1.

**Table 1:** Effect of heavy metals on embryonic and larval development of fish.

**Species**	**Dose**	**Exposure period**	**Alterations/ type of deformities**	**References**
Cd				
*Odontesthes bonariensis*	0.25, 2.5 µg/l	10 days	Reduced embryo and larval survivability	[41]
*Oncorhynchus mykiss*	2 µg/l	4 days	Larval erythroblasts with MN, NB and BN	[52]
*Danio rerio*	60 ppb	7 dpf	Decreased diameter of the saccule otolith, otoliths with numerous fiber between knobs	[53]
*Cyprinus carpio*	0.3, 0.06 mg/l	60 days	Lowest survival and growth rate, malformation in the yolk sac, curvature in vertebral column, body shortening, and cardiac edema	[49]
*Leuciscus idus*	0.1 mg/l	21 dah	Lowest survival, body length, body perimeter area, swim bladder	[35]
*Oryzias latipes*	0.18–19.8 μg/l	10 days	Spinal deformities (kyphosis, lordosis and C-shaped larvae)	[47]
*Silurus soldatovi*	0.0001–30 mg/l	144 h	Spinal curvature	[34]
*Gambusia affinis*	0.4 mg/l	30 days	Spinal (kyphosis, lordosis and scoliosis)	[46]
*Pagrus major*	0–3.2 mg/l	-	Cardiac edema, blastodermal lesions and skeletal deformities (spinal curvature, degenerated and hooked tails, fins lesions)	[32]
*Rhamdia quelen*	0.0005–0.018 mg/l	21 dah	Deformed spinal column	[50]
*Oncorhynchus mykiss*	0.05, 0.25, 0.50 & 2.50 μg/l	56 days	Premature hatching, delayed hatching, lower larval growth	[54]
*Danio rerio*	3.3, 6.7 & 13.3 μM	80 hpf	Edema (pericardial, yolk sac), decreased pericardial area and length of tail, lordosis	[55]
*Cyprinus carpio*	0.2 mg/l	30 days	Growth retardation	[56]
*Clarias gariepinus*	0.05–5.00 mg/l	5 days	Reduction of pigmentation, 100% mortality in 1.5 and 5.0 mg/l	[40]
*Cyprinus carpio*	5–50 mg/l	-	Swelling of eggs with increasing concentration	[57]
*Melanotaenia fluviatilis*	0.033–3.3 mg/l	2 h	Spinal abnormalities	[58]
Cr				
*Odontesthes bonariensis*	4, 40 µg/l	10 days	Reduced embryo and larval survivability, morphological alteration (C-shaped body)	[41]
*Danio rerio*	50, 500 mg/l	4 days	Increased embryo mortality and heart rate of the hatched eggs	[51]
*Clarias gariepinus*	11–114 mg/l	5 days	Abnormal body axis, reduced larval survivability and growth	[40]
Cu				
*Oryzias melastigma*	0.32 mg/l	7 days	Skeletal and vascular system abnormalities (anemia, hemorrhage), reduction of pigmentation, absence of eye	[11]
*Odontesthes bonariensis*	22, 220 µg/l	10 days	Reduced embryo and larval survivability	[41]
*Danio rerio*	50, 500 mg/l	4 days	Increased embryo mortality and heart rate of the hatched eggs	[51]
*Leuciscus idus*	0.1 mg/l	21 days	Vertebral curvatures, yolk sac deformities, shorten body length, body perimeter area, swim bladder perimeter area	[35]
*Carassius auratu*	0.1–1 mg/l	24 hah	Scoliosis and tail curvatures	[44]
*Oryzias latipes*	6.95–23.1 μg/l	10 days	Spinal deformities (kyphosis and lordosis), yolk-sac mal-absorption, abnormal cardiovascular system	[47]
*Fundulus heteroclitus*	0.0005–0.004 mg/l	50 days	Vertebral deformities and inflammatory masses	[59]
*Oncorhynchus mykiss*	0.22 mg/l	4 days	Increased mortality of embryos	[48]
*Danio rerio*	0.068-0.244 mg/l	120 haf	Lateral line deformities (fewer functional neuromasts)	[31]
*Danio rerio*	50-1000 μg/l	3 dpf	Low hatching rate, higher heart rate, larger yolk sac	[31]
*Cyprinus carpio*	0.2 mg/l	-	First developmental retardation, Retardation of hatching	[60]
*Cyprinus carpio*	0.2 mg/l	20 day	Curvature of the spine, C-shaped larva, deformed yolk sac, shortened body	[61]
*Cyprinus carpio*	0.2 mg/l	30 days	Growth retardation	[56]
*Clarias gariepinus*	0.15–2.5 mg/L	5 days	Reduction of pigmentation	[40]
*Cyprinus carpio*	2 mg/l	-	Larvae with axial and lateral curvatures of spine, C shaped larvae, eye anomalies, deformed yolk sac, cardiac edema	[62]
Hg				
*Danio rerio*	20 and 30 mg/l	-	Abnormal fin, flexure of the posterior tail region	[38]
Pb				
*Clarias gariepinus*	0.1–0.5 mg/L	48–168 h	Irregular head, notochord defects, yolk-sac edema, spinal curvatures etc.	[42]
Zn				
*Odontesthes bonariensis*	211, 2110 µg/L	10 days	Cumulative embryo survival was significantly reduced to 40% at day 6 and 10% at day 2 respectively	[41]
*Danio rerio*	50, 500 mg/l	4 days	Majority of eggs were dead within 48 hr because of its severe toxicity, the heart rate of the hatched eggs increased with increasing concentration	[51]
*Pagrus major*	0.1, 0.3, 0.5, 0.7, 1.0, 1.5, 2.0, 2.5 mg/l	10 days	Low hatching rate, high mortality, abnormal pigmentation, hooked tail, spinal deformity, pericardial edema, and visceral hemorrhage	[33]
*Oncorhynchus mykiss*	0.3 mg/l	4 days	Increased mortality of embryos	[48]
*Melanotaenia fluviatilis*	0.33–33.3 mg/l	2 h	Spinal deformities	[58]

MN; micronucleus, NB; nuclear bud, BN; bi-nucleated

Most of the literature reported reduced embryonic and larval survival, reduced and delayed hatching, stunted growth rate and morphological abnormalities such as skeletal deformities, vascular system abnormalities, reduction in pigmentation, eye anomalies etc. among different fish species exposed to lethal and sub-lethal doses of essential (Cu, Zn) and non-essential (Cd, Cr, Hg and Pb) heavy metals [32], [38], [40], [41], [42]. Cardiovascular endpoints such as hyper or hypo dystrophia, positioning abnormality, incomplete or abnormal heart looping, tubular heart, oedemata, megalocardia etc. are important parameters to assess the toxicity of heavy metals in embryos and larvae, revealing species-dependent differences in the responses to various heavy metals. For example, Cu exposure significantly increased heart rate in zebrafish embryo [31], whereas cardiac activity is reduced in red sea bream [32] and zebrafish [43] embryos exposed to Cd. Larvae are less tolerant to heavy metals than the embryo since embryos have protective hard chorion layers and perivitelline fluid that can impede the entry of heavy metals [44], [45]. Catalase (CAT, the enzyme which converts relatively toxic hydrogen peroxide to oxygen activity is significantly reduced in the larvae compared to embryos, which might contribute to the resistance of embryos to heavy metals.

Toxicity levels of heavy metals in embryos and larvae of freshwater fish are different from marine fish because of salinity differences. At higher salinity levels, the bioavailability of the toxic forms of heavy metals in water decreases. Information is limited about the toxic effects of heavy metals on marine fish embryos and larvae. Low hatchability, high mortality, morphological abnormalities etc. are reported in embryos and larvae of marine fish exposed to different heavy metals [11], [32]. Environmental cues especially high temperature is known to cause developmental deformities in fishes and it has been reported that combined application of high temperature (24-32⁰C) and heavy metal such as Cd causes intense increase in skeletal deformities in juvenile mosquito fish (*Gambusia affinis*) than Cd or temperature alone [46]. High temperature increases the metabolic activity of fish, increasing the potentiality of metal ion action (Cd in this case) on cellular enzyme and cell membrane.

The mode of action (especially changes in enzyme and DNA) of each heavy metal exposure in embryo and larvae are at early stage of investigation and gaining importance among the researchers investigating molecular mechanisms of their effects in fish. Superoxide dismutase (SOD) and catalase (CAT) enzymes are known to convert reactive oxygen species to non-toxic oxygen in the liver. It has been found that in embryos and larvae of goldfish (*Carassius auratus*), these enzymatic activities were significantly inhibited due after exposure of high Cu concentration (1.0 mg/L), causing oxidative stress responsible for lipid peroxidation [44]. Moreover, Cd and Cu exposure to 2 dph larvae of Japanese medaka (*Oryzias latipes*) induced significant DNA damage [47] determined by Comet assay (a reliable method to assess genotoxicity in all stages of fish).

There are numerous reports on the effect of single heavy metal on the ontogenic development embryos and larvae. Because most of the open water environment is contaminated with mixtures of heavy metals (from anthropogenic and geogenic sources), it is important to evaluate the combined effects of those heavy metals on embryonic and larval development. The combined effect of Cu-Zn and Cd-Zn has been investigated in Rainbow trout *Oncorhynchus mykiss* [48] and common carp *Cyprinus carpio* [49] embryos respectively, revealing increased embryonic mortality and physical deformities (e.g. vertebral column deformities). Hg and Pb toxicity resulted defects of important organs of fish such as abnormal and irregular fins, head, tails and several spinal difficulties [38], [42]. Moreover, Zn contamination negatively affected the hatching success and survival of several fish species as well as hampered the normal formation and pigmentation of several organs [33], [35], [41], [48].

Supplementation of vitamin C with the dry feed to the embryo and larvae of common carp (*Cyprinus carpio*) exposed to mixture of Zn and Cd increased the ontogenic development and quality and quantity of the larvae through the improvement of immune system [49]. It has been reported that Cd exposure under conditions of high alkalinity can significantly increase the hatching, survival rate and growth of larvae of Silver catfish *Rhamdia quelen* [50].

## 3 Impact of heavy metals on growth performance of fish

**Fig. 1:**
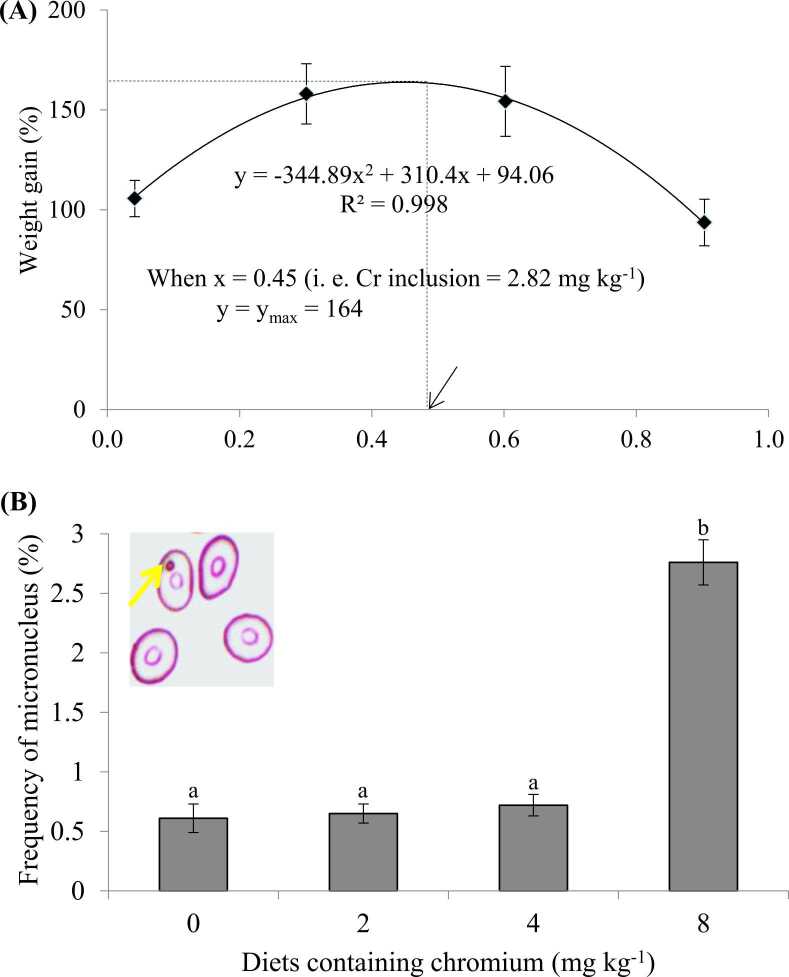
Effects of dietary Cr on (A) weight gain (WG) and (B) frequency of formation of micronucleus (MN) in the erythrocytes of striped catfish. The analyzed dietary Cr concentration was log transformed for better visualization. Requirement derived with the polynomial regression method for WG was 2.82. Values with different alphabetical superscripts differ significantly (p < 0.01) among different diets.

Nutritional adequacy is prerequisite sustainable aquaculture. The overall growth, health status and reproductive performances of various aquaculture species especially fish are dependant on appropriate nutrition [63], [64], [65]. Among the various candidates that contribute nutritional demand of various aquaculture species, heavy metals play important roles in this regard. Various types of trace metals significantly contribute to different physiological processes including growth of fish (Table 2). Several trace metals such as Mn, Fe, Co, Cu, Cr and Zn are known to be important minerals with positively influences on the physiology and metabolism of fish [9], [10]. Cr has been regarded as very important trace element that improved the health status of several animals through upgrading the physiology as well as their metabolism [66], [67]. Cr directly involved in nutrient (protein, lipid and carbohydrates) metabolism significantly influences the growth and feed utilization of several fish species [68], [69]. Moreover, Cr also altered the fatty acid profile in blood through participating in fatty acid metabolism in various animals [70], [71]. It has been found that Cr supplementation lowered the cholesterol, triglycerides level in blood and increased the high density lipoprotein (HDL) cholesterol level [72], [73]. Dietary Cr significantly influenced the expression of several genes related to glucose metabolism, lipogenesis, apparently playing a key role in growth enhancement [74]. Cr supplementation in diet significantly improved the growth and feed utility of striped catfish (*Pangasianodon hypophthalmus*) upto 4 mg/kg but greater concentrations resulted in lower growth with higher micronucleus frequencies (Fig. 1) [10]. On the contrary, presence of Cr in excess level led to several toxicities and therefore, reduced the growth and feed palatability of several species [75], [76], [77]. Zn is an essential trace element that plays a significant role in the life processes of animals including fish [78], [79], [80]. Zn acts as a co-factor of several metallo-enzymes (carbonic anhydrase, alkaline phosphatase, alcohol dehydrogenase etc.) ensuring the availability and activities of those important enzymes to stimulate digestion and metabolism of nutrients [81], [82], [83]. Zn also regulates the nucleic acid metabolism, protein synthesis and anti-oxidative enzymes functionalities of fish [84]. The anti-oxidative roles of Zn were well demonstrated in several studies [85], [86]. Dietary Zn supplementation considerably improved the growth of fish through upgrading muscle morphology [9]. Dietary Zn provisions also influence the whole body composition of fish muscle. Zn significantly enhanced the lipid content and lowered the moisture and ash level of fish carcass [87]. However, Zn deficiency hampers the nucleic acid and protein biosynthetic pathways [66], [88], impairment of bone development [87] and various other pathological effects [89]. On the other hand, excess amount of Zn resulted various negative impacts such as growth and reproductive performance reduction [90], oxidative stress [91] and poor feed utilization [92], [93], [94]. Moreover, Zn toxicity resulted in delayed hatching, malformations in bone calcification and growth defects [95]. Cu is an essential element that plays a pivotal role in various physiological as well as biological systems such as hemoglobin and bone formation, control the activities of myelin in the nervous system and finally acts as an activists of many important enzymatic action including cytrochrome oxidase, lysyl oxidase, dopamine hydroxylase ferroxidase, tyrosinase and Cu-Zn superoxidase dismutase [93], [96]. Various studies revealed that dietary Cu supplementation significantly improve the growth, oxidative status and immune system of several aquatic species [96], [97], [98], [99]. In the very recent years, aquaculture nutritionists find out the outstanding role of Cu particles has caught the attention aquaculture personnel as potentially interesting feed supplement [100], [101]. On the contrary, dietary Cu toxicity exhibited several adverse effects including reduced growth, greater FCR, lower feed efficiency [102], [103]. Fe, an essential element that helps to maintain the normal activities of different organs and tissues of animals including fish because of its active role in physiological processes like oxygen gas transportation, cellular respiratory activities, and lipid peroxidation processes. Fe modulated the immune system of animals and thus protects against various infectious agents and also actively participates in the synthesis of steroid and DNA, drug metabolism and electron transportation [104].

**Table 2:** Impacts of heavy metals on growth performance of fish.

**Species**	**Doses (mg/kg)**	**Exposure time (days)**	**Effects**	**References**
**As**				
*Oncorhynchus mykiss*	26–77 µg/kg	30	Growth reduced accompanied by slower feeding rate, reduced FCE	[105]
Cd				
*Mystus seenghala*	1/3 of LC_50_	112	Lowered average wet weight, body length and condition factor while higher FCR	[106]
*Ictalurus punctatus*	0.5, 2, 6 μg/L	180	Negatively impacted on growth (length and weight)	[107]
*Pelteobagrus fulvidraco*	0, 50 and 200 μg/L	56	Growth retardation; decreased WG and SGR in both 50 and 200 μg/L	[108]
*Oreochromis niloticus*	0, 25, 50	84	Lowest BW and WG at 50 mg/kg	[109]
*Danio rerio*	30 µg/L	35	Reduced growth and survival rate	[110]
*Danio rerio*	30 μg/l	35	Inhibited body weight, SGR and survival rate	[111]
*Oreochromis niloticus*	0.5	56	Reduced growth and feed intake	[112]
*Oncorhynchus mykiss*	1 and 3 μg/l	30	Condition Factor (K), SGR, BWG decreased, while FCR increased	[113]
*Ctenopharyngodon idella*	0, 5, 500 µg/l	56	Reduction in growth	[114]
*Pelteobagrus fulvidraco*	0.25, 4.92, 48.57, 474.7	28	WG, SGR, FI, PER declined with increasing dietary Cd	[115]
Cr				
*Pangasianodon hypophthalmus*	2, 4, & 8	60	The growth and feed utilization increased significantly in the fish fed with 2 and 4 mg/kg supplemented diets	[10]
*Labeo rohita*	0.4, 0.8 & 1.2	60	Improved %WG, SGR, FER and PER and %ANPU at 0.8 mg kg^-1^	[116]
*Oreochromis niloticus*	4.57 mg/L	60	WG, SGR reduced	[117]
*Platichthys stellatus*	0, 50, 100, 200, 400 ppb	28	DLG, DWG, CF, and HSI decreased	[118]
*Megalobrama amblycephala*	0.2, 0.4, 0.8, 1.6, 3.2 & 12.0	77	Highest FW and SGR; lowest FCR in fish fed with 0.4 mg/kg	[119]
*Sebastes schlegelii*	0, 30, 60, 120 & 240	28	Decreased growth performance	[120]
*Larmichthys crocea*	5, 10, 20, 40 & 80	70	Higher survival and SGR in fish fed the diet with 5 mg/kg	[101]
*Cyprinus carpio*	0.5, 1.0, 2.0	56	produced superior %WG, SGR, FCR and PER at a level 0.5 mg/kg	[121]
*Oreochromis niloticus*	200, 400, 600, 800, 1000 & 1200 ppb	72	increased FI at 400 ppb and 600 ppb	[122]
*Cyprinus carpio*	0.5, 1.0, 2.0	63	higher FBW, %WG, SGR and lower FCR at 0.5 mg/kg	[121]
*Ctenopharyngodon idellus*	0.2, 0.4, 0.8, 1.6 & 3.2	70	improved WG, FER, PER and PR at 0.8 mg kg^-1^	[123]
*Channa punctatus*	2 & 4	60	BWG was comparatively less in fish exposed to 4 mg/L than the 2 mg/L and control	[124]
Cu				
*Cyprinus carpio*	0.05 & 0.1	90	Significantly reduced SGR, WG, PER and increased FCR	[125]
*Megalobrama amblycephala*	1.43 & 9.13	70	Improved growth performance	[126]
*Oreochromis niloticus*	25, 50 & 75 µg/L	90	Decrease in FW, WG, and HSI	[127]
*Cyprinus carpio*	0, 1.5 & 3.0	60	Decrease in WG, length, CF and increase in FCR	[128]
*Poecilia vivipara*	5 & 9 μg/L	365	Exposure to 9 μg/L Cu reduced fish body weight and length	[129]
*Pagrus major*	2	60	Increased FBW, WG, SGR, FI, FER, PER, PG and PR	[97]
*Pagrus major*	2, 4, 6, 8	60	Highest final body weight, WG, SGR, FI, protein gain at levels of 2 and 4 mg/kg	[97]
*Channa punctatus*	3.7, 4.7, 5.7, 6.7, 7.7 & 8.7	84	Fish fed diet with 6.7 mg kg^−1^ copper had highest AWG, PER, PG and best FCR	[130]
*Cyprinus carpio*	20, 30, 40 & 70 µg/l	28	Decrease in TL, WG and CF, and increase in HSI	[131]
*Carassius carassius*	0.30 & 0.60	20	High-concentration (0.60 mg/L) hindered the growth	[132]
*Poecilia reticulata*	0, 0.004, 0.013, 0.019, 0.029	56	Decrease in FW, SGR, and increase in FCR	[133]
*Lateolabrax japonicus*	0 & 4	56	Higher FI, SGR, PER	[100]
*Huso huso*	1.1, 3.5, 7.1, 9.7, 13.1, 25.1, 49.9 & 195	84	Weight gain of fish fed 10 and 13 mg/kg diets was higher than others.	[96]
*Ctenopharyngodon idella*	2.26, 3.75, 5.25, 6.70 & 8.33	56	increased %WG and FI at up to 3.75 mg/kg	[134]
*Ctenopharyngodon idella*	2.26, 3.75, 5.25, 6.70 & 8.33	56	increased %WG and FI at up to 3.75 mg/kg	[134]
*Ctenopharyngodon idella*	2.26, 3.75, 5.25, 6.70, & 8.33	56	PWG and FI increased with dietary Cu levels up to 3.75 mg/kg	[134]
*Megalobrama amblycephala*	0, 3, 6, 9, 25, 50, 100 & 150	56	Higher WG, SGR in fish fed diets supplemented with 3–6 mg/kg	[135]
*Synechogobius hasta*	0, 0.15 & 0.3	15	WG and SGR declined	[67]
*Sebastes schlegeli*	0, 50, 125, 250 & 500	60	reduced the growth rate	[136]
*Oncorhynchus mykiss*	35.7 & 54.1 μg/l	56	fish exposed to higher Cu concentrations growing slower	[137]
Fe				
*Clarias gariepinus*	0.2, 0.4, 0.8, 1.2 & 1.6	49	Improved WG, %WG, SGR, FCR in fish fed the Fe supplemented diet	[138]
*Ctenopharyngodon idella*	12.15, 35.38, 63.47, 86.43, 111.09, 136.37	60	FBW, PWG, SGR and FI increased significantly up to 207 63.47 mg/kg diet and then decreased significantly	[139]
*Cyprinus carpio*	53.9, 90.0, 115.6, 146.1, 176.0, 215.8 & 266.0	60	Improved %WG, FE, PER in fish fed the diet up to 90.0 mg/kg	[140]
*Epinephelus coioides*	0, 50, 100, 150, 200 &250	56	highest WG and FE in fish fed the diet supplemented with 100 mg/kg	[141]
*Ictalurus puctatus*	40, 336 & 671	70	Best growth at 40 and 336 mg/kg diet	[142]
*Ictalurus punctatus*	0, 30 & 300	112	Increased WG and survival; better FCR in fish fed the diet up to 300 mg/kg	[143]
Zn				
Oreochromis niloticus	80	42	Improved growth parameters (WG, %WG, and SGR) and feed utilization (FCR and PER)	[9]
*Cyprinus carpio*	15.3, 26.9, 40.8, 58.2, 68.9 & 92.5	42	Enhanced %WG, FE, PER and LPV with dietary levels up to 40.8 mg/kg	[93]
*Salmo salar*	50, 180	180	Increased SGR at higher concentration, better FCR	[144]
Pb				
*Chanos chanos*	0, 42.64, 63.97 & 85.2	40	WG, LG, SGR, FE, and FCR declined significantly at the highest concentration	[145]
*Catla catla,*				
*Labeo rohita*				
*Cirrhina mrigala*	1/3rd of LC50	60	Lesser WG, FI and FCE	[146]

ANPU; apparent net protein utilization, FCR; feed conversion ratio, LPV; lipid productive value, FE; feed efficiency, FER; feed efficiency ratio, PER; protein efficiency ratio, FBW; final bodyweight, WG; weight gain, SGR; specific growth rate, FI; feed intake, FER; feed efficiency ratio, PER; protein efficiency ratio, PG; protein gain, PR; protein retention

## 4 Heavy metals effect on reproduction of fish

Reproduction is essential to all animals and successful reproductive performance among the most important determinants of survival at the species level [147], [148], [149]. Heavy metals pollution negatively affects the reproductive performance of fish resulting low quality gametes that may influence not only success rate of fertilization but also hatching as well as survival rate of the offspring (Table 3) [150]. Various types of heavy metals accumulated into the fish body from the environment and their continuous accumulation disrupt the formation and activities of various tissues and organs including reproductive organs [62]. Heavy metals caused anomalies in reproductive cell/organ development. Arsenic (As) pollution seriously affected the reproductive performances of fish through inhibition of spermatogenesis and oogenesis including reduced egg and sperm quality and quantity, hatching and fertilization rate [22], [23], [24]. Cd is a potent hazardous metal that resulted several dysfunctions of reproductive process of fish. Various studies demonstrated several difficulties in reproductive performance of fish such as abnormal oocytes structure, empty follicle and loosing follicular line, retraction as well as condensation of cytoplasm, total GSI reduced and so on [27]. Moreover, Cd toxicities cause shrinkage of spermatic lobules and fibrosis in testis, lower sperm motility and viability as well as reduced fertilization rate [26], [150], [151], [152], [153]. Cr has been regarded as one of the most biologically potent heavy metals due to its summative destructive effects on living organisms [154]. Long term exposure to Cr drastically reduced the spawning success [155], fibrotic and pyknotic testis [26], significantly reduced the GSI, fecundity, lowered number of oocytes and matured spermatozoa [156], hampered the motility of sperm [150] and finally gradual decrease of vitellogenic oocytes [124]. Various studies revealed that reduced GSI, fecundity, hatching rate, fertilization success, abnormal shape of reproductive organs, and finally overall reproductive success resulted from the toxicities created by Cu and Hg [28], [29], [151], [157], [158], [159], [160]. Pd and Zn resulted similar deformities as well as negative impacts in *Carassius gibelio* [30], *Odontesthes bonariensis* [26]; *Oryzias melastigma* [27] and *Clarias magur* [25].

**Table 3:** Effects of heavy metals on reproductive performances of fish.

Fish species	Doses	Exposure period (days)	Effects	References
As				
*Anguilla japonica*	0.1, 100 μM	15	Inhibited spermatogenesis via steroidogenesis suppression	[24]
*Danio rerio*	-	68	Reduced reproductive output, egg production, number of spawns, average number of eggs per spawn and hatching rate	[23]
*Anguilla japonica*	10^−5^ M	6	Inhibited the spermatogenesis, necrosis of testicular fragments	[22]
Cd				
*Oryzias melastigma*	10 μg/L	30	irregular oocytes, partly adhesion, empty follicle, and increased follicular atresia, cytoplasmic retraction, cytoplasm condensed form, karyoplasm clumping, loose follicular lining	[27]
*Gasterosteus aculeatus*	1 µg/L	90	GSI decreased in prolonged exposure	[161]
*Odontesthes bonariensis*	0.25 μg/L	14	Testis showed fibrosis and shrinkage of the spermatic lobules, pyknotic cells, reduce of the length of the spermatic lobules	[26]
*Cyprinus carpio*	50, 100, 150 & 200 ppm	3	Sperm quality (motility and viability) and fertilization rate decreased at 100 ppm or more	[153]
*Acipenser baerii*	0–100 mg/L	4 h	Percentage of motile sperm was reduced from 10 mg/l to higher conc.	[151]
*Oncorhynchus mykiss*	10, 100 and 500 mg/l	4 h	Altered sperm motility characteristics and hatching rates	[152]
*Acipenser ruthenus*	0.1, 5.0 mg/L	2 h	Sperm motility parameters (motility and velocity) inhibited in higher conc.	[150]
Cr				
*Oryzias melastigma*	½ of 96LC50	60	After long-term exposure amount of spawning decreased	[155]
*Odontesthes bonariensis*	4 μg/L	14	Testis showed fibrosis and shrinkage of the spermatic lobules, pyknotic cells in the testis	[26]
*Oryzias latipes*	4 mg/L	90	Decreases in gonad weight, GSI and fecundity, reduced number of mature oocyte and mature spermatozoa in testes	[156]
*Acipenser ruthenus*	0.1, 5.0 mg/L	2 h	Sperm motility parameters (motility and velocity) inhibited in higher conc.	[150]
*Channa punctatus*	4 mg/L	30	Decreased the percentage of vitellogenic oocytes	[124]
Cu				
*Poecilia reticulata*	0, 5, 10 mg/L	56	Lowest reproductive success, prolonged parturition time and highest mortality rate at 10 mg/l	[28]
*Oreochromis niloticus*	1, 2, 4 mg/kg	4	Decrease in sperm motility rate, VCL, VAP, and VSL,	[29]
*Odontesthes bonariensis*	22 μg/L	14	Fibrosis and shrinkage of the spermatic lobules, pyknotic cells in the testis, reduce of the length of the spermatic lobules	[26]
*Xiphophorus helleri*	0.04, 0.08, 0.12 & 0.16 ppm	100	Decreased GSI, gonad not developed in high concentrations (0.12 and 0.16 ppm)	[160]
*Carassius auratus*	0.25, 0.05, 0.075 & 0.1 ppm	100	Decreased GSI, reduced the fecundity	[160]
*Danio rerio*	100, 500 & 1000 μg/g	260	1000 μg produce decrease in GSI but not significant.	[159]
Hg				
*Acipenser baerii*	0-100 mg/L	4 h	Percentage of motile sperm reduced from 1 mg/l to higher conc and complete obstruction in 100 mg/l.	[151]
*Oncorhynchus mykiss*	1, 10, 100 mg/l	4 h	Inhibition of sperm motility	[152]
*Dicentrarchus labrax*	0.01, 0.1, 1, 10 & 100 ppm	-	Exposure to100 ppm completely inhibited sperm motility	[158]
*Oryzias latipes*	40 μg/L	8	Testicular atrophy and arrested spermiation	[157]
*Pimephales promelas*	0.87 to 3.93 μg/g diet	250	Lowered GSI, Reduced the reproductive success	[162]
goldfish	1, 10 & 100 μg/L	-	Reduced curvilinear velocity, percentage of motile sperm, and flagella length	[163]
*Pimephales promelas*	0.88, 4.11 & 8.46 µg/g	-	Delayed spawning, and days to spawning Reduced the instantaneous rate of reproduction, GSI and reproductive efforts	[43]
*Oreochromis niloticus*	0.08 to 0.54 μg/g	210	The normal morphology of the testes was altered, Decreased spermatogenesis	[164]
Pb				
*Oryzias melastigma*	50 μg/L	30	Irregular oocytes, partly adhesion, empty follicle, increased follicular atresia, loose follicular lining	[27]
*Carassius gibelio*	8, 13, 24 & 49 mg/kg	365	Decreased GSI, affected ovarian steroidogenesis, gametogenesis, ovulation	[30]
Zn				
*Clarias magur*	50, 200, 300 mg/kg	60	The highest GSI and fecundity at 50 mg/l	[25]
*Oryzias melastigma*	100 μg/L	30	Irregular oocytes, partly adhesion, empty follicle, and increased follicular atresia, loose follicular lining	[27]
*Odontesthes bonariensis*	211 μg/L	14	Fibrosis and shrinkage of the spermatic lobules, pyknotic cells in the testis, reduced the length of the spermatic lobules,	[26]
*Cyprinus carpio*	10, 50, 100, 200, 500, 1000 and 2000 ppm	-	Decreased the motility of sperm, inhibitory influence on VSL, low fertilization rate	[165]

GSI; gonad-somatic index,

## 5 Conclusion and future perspectives

Heavy metals contamination is a serious threat to entire aquatic ecosystems including associated flora and fauna. The devastating impacts of heavy metals on aquatic organisms specifically fish result an irreparable loss in aquaculture industry. In this review, destructive effects of heavy metals on fish focusing the embryonic and larval development, growth and reproduction of commercially important species are discussed very concisely with a view to using it as a tool for further genotoxicity related experiments by the researchers of the associated areas. Heavy metals resulted in severe deformities in several aquatic organisms that will ultimately pose a substantial threat to associated consumers. To enlarge the sustainability of the aquaculture sector and to produce safe fish for human consumption, regular monitoring of the fish and associated environment should be done by the appropriate authorities at the local government, state, and national levels. A well-established framework should be developed as soon as possible to mitigate this great problem.

## CRediT authorship contribution statement

Khanam Taslima: preparation of the first draft of the manuscript. Md Al-Emran, Mohammad Shadiqur Rahman, Jabed Hasan, Zannatul Ferdous and Md Fazle Rohani: data collection and preparation of the Tables. Md Shahjahan: conceptualization, edited the manuscript and final approval. All authors have read the final version and approved the manuscript.

## Declaration of Competing Interest

The authors declare that they have no known competing financial interests or personal relationships that could have appeared to influence the work reported in this paper.

## Data Availability

Sharing of data is not permissible for this article. The data that support the outcomes of this study are available on request from the corresponding author [M Shahjahan].

## References

[1] Sarkar M., Islam J.B., Akter S. (2016). Pollution and ecological risk assessment for the environmentally impacted Turag River, Bangladesh. J. Mater. Environ. Sci..

[2] Mahmuda M., Rahman M.H., Bashar A., Rohani M.F., Hossain M.S. (2020). Heavy metal contamination in tilapia, *Oreochromis niloticus* collected from different fish markets of Mymensingh District. J. Agric. Food Environ.

[3] Ezemonye L.I., Adebayo P.O., Enuneku A.A., Tongo I., Ogbomida E. (2019). Potential health risk consequences of heavy metal concentrations in surface water, shrimp (Macrobrachium macrobrachion) and fish (*Brycinus longipinnis*) from Benin River, Nigeria. Toxicol. Reports.

[4] Sarkar M.M., Rohani M.F., Hossain M.A.R., Shahjahan M. (2021). Evaluation of heavy metal contamination in some selected commercial fish feeds used in Bangladesh. Biol. Trace Elem. Res..

[5] Shahjahan M., Islam S.M., Bablee A.L., Siddik M.A.B., Fotedar R. (2021). Sumithion usage in aquaculture: benefit or forfeit?. Rev. Aquac..

[6] Shahjahan M., Rahman M.S., Islam S.M.M., Uddin M.H., Al-Emran M. (2019). Increase in water temperature increases acute toxicity of sumithion causing nuclear and cellular abnormalities in peripheral erythrocytes of zebrafish *Danio rerio*. Environ. Sci. Pollut. Res..

[7] Abdel-Baki A.S., Dkhil M.A., Al-Quraishy S. (2011). Bioaccumulation of some heavy metals in tilapia fish relevant to their concentration in water and sediment of Wadi Hanifah, Saudi Arabia. African J. Biotechnol..

[8] Fernandes C., Fontaínhas-Fernandes A., Cabral D., Salgado M.A. (2008). Heavy metals in water, sediment and tissues of *Liza saliens* from Esmoriz-Paramos lagoon, Portugal. Environ. Monit. Assess..

[9] Rohani M.F., Bristy A.A., Hasan J., Hossain K., Shahjahan M. (2021). Dietary zinc in association with vitamin E promotes growth performance of Nile tilapia. Biol. Trace Elem. Res..

[10] Akter S., Jahan N., Rohani M.F., Akter Y., Shahjahan M. (2021). Chromium supplementation in diet enhances growth and feed utilization of striped satfish (*Pangasianodon hypophthalmus*). Biol. Trace Elem. Res..

[11] Wang R.F., Zhu L.M., Zhang J., An X.P., Yang Y.P., Song M., Zhang L. (2020). Developmental toxicity of copper in marine medaka (*Oryzias melastigma*) embryos and larvae. Chemosphere..

[12] Saffari S., Keyvanshokooh S., Zakeri M., Johari S.A., Pasha-Zanoosi H., Mozanzadeh M.T. (2018). Effects of dietary organic, inorganic, and nanoparticulate selenium sources on growth, hemato-immunological, and serum biochemical parameters of common carp (*Cyprinus carpio*). Fish Physiol. Biochem..

[13] Song Z.-X., Jiang W.-D., Liu Y., Wu P., Jiang J., Zhou X.-Q., Kuang S.-Y., Tang L., Tang W.-N., Zhang Y.-A., Feng L. (2017). Dietary zinc deficiency reduced growth performance, intestinal immune and physical barrier functions related to NF-κB, TOR, Nrf2, JNK and MLCK signaling pathway of young grass carp (*Ctenopharyngodon idella*). Fish Shellfish Immunol.

[14] Dawood M.A.O., Zommara M., Eweedah N.M., Helal A.I. (2020). The evaluation of growth performance, blood health, oxidative status and immune-related gene expression in Nile tilapia (*Oreochromis niloticus*) fed dietary nanoselenium spheres produced by lactic acid bacteria. Aquaculture..

[15] Ghazi S., Diab A.M., Khalafalla M.M., Mohamed R.A. (2021). Synergistic effects of selenium and zinc oxide nanoparticles on growth performance, hemato-biochemical profile, immune and oxidative stress responses, and intestinal morphometry of nile tilapia (*Oreochromis niloticus*). Biol. Trace Elem. Res..

[16] Okoye P.A.C., Ajiwe V.I.E., Okeke O.R., Ujah I.I., Asalu U.B., Okeke D.O. (2015). Estimation of heavy metal levels in the muscle, gizzard, liver and kidney of broiler, layer and local (cockerel) chickens raised within awka metropolis and its environs, anambra state, south eastern Nigeria. J. Environ. Prot..

[17] Mokarram M., Saber A., Sheykhi V. (2020). Effects of heavy metal contamination on river water quality due to release of industrial effluents. J. Clean. Prod..

[18] Suchana S.A., Ahmed M.S., Islam S.M.M., Rahman M.L., Rohani M.F., Ferdusi T., Ahmmad A.K.S., Fatema M.K., Badruzzaman M., Shahjahan M. (2021). Chromium exposure causes structural aberrations of erythrocytes, gills, liver, kidney, and genetic damage in striped catfish *Pangasianodon hypophthalmus*. Biol. Trace Elem. Res..

[19] Islam S.M.M., Rohani M.F., Zabed S.A., Islam M.T., Jannat R., Akter Y., Shahjahan M. (2020). Acute effects of chromium on hemato-biochemical parameters and morphology of erythrocytes in striped catfish *Pangasianodon hypophthalmus*. Toxicol. Reports..

[20] Islam S.M.M., Rahman M.A., Nahar S., Uddin M.H., Haque M.M., Shahjahan M. (2019). Acute toxicity of an organophosphate insecticide sumithion to striped catfish *Pangasianodon hypophthalmus*. Toxicol. Reports..

[21] Ahmed M.K., Habibullah-Al-Mamun M., Parvin E., Akter M.S., Khan M.S. (2013). Arsenic induced toxicity and histopathological changes in gill and liver tissue of freshwater fish, tilapia *Oreochromis mossambicus*. Exp. Toxicol. Pathol..

[22] Yamaguchi S., Miura C., Ito A., Agusa T., Iwata H., Tanabe S., Tuyen B.C., Miura T. (2007). Effects of lead, molybdenum, rubidium, arsenic and organochlorines on spermatogenesis in fish: monitoring at Mekong Delta area and in vitro experiment. Aquat. Toxicol..

[23] Boyle D., Brix K.V., Amlund H., Lundebye A.K., Hogstrand C., Bury N.R. (2008). Natural arsenic contaminated diets perturb reproduction in fish. Environ. Sci. Technol..

[24] Celino F.T., Yamaguchi S., Miura C., Miura T. (2009). Arsenic inhibits in vitro spermatogenesis and induces germ cell apoptosis in Japanese eel (*Anguilla japonica*). Reproduction..

[25] Gupta G., Srivastava P.P., Kumar M., Varghese T., Chanu T.I., Gupta S., Ande M.P., Jana P. (2021). The modulation effects of dietary zinc on reproductive performance and gonadotropins’ (FSH and LH) expression in threatened Asian catfish, *Clarias magur* (Hamilton, 1822) broodfish. Aquac. Res..

[26] Gárriz Á., del Fresno P.S., Carriquiriborde P., Miranda L.A. (2019). Effects of heavy metals identified in Chascomús shallow lake on the endocrine-reproductive axis of pejerrey fish (*Odontesthes bonariensis*). Gen. Comp. Endocrinol..

[27] Yan W., Hamid N., Deng S., Jia P.P., Pei D.S. (2020). Individual and combined toxicogenetic effects of microplastics and heavy metals (Cd, Pb, and Zn) perturb gut microbiota homeostasis and gonadal development in marine medaka (*Oryzias melastigma*). J. Hazard. Mater..

[28] Forouhar Vajargah M., Mohamadi Yalsuyi A., Sattari M., Prokić M.D., Faggio C. (2020). Effects of copper oxide nanoparticles (CuO-NPs) on parturition time, survival rate and reproductive success of guppy fish, *Poecilia reticulata*. J. Clust. Sci..

[29] Santos G.S., Neumann G., do Nascimento C.Z., Domingues C.E., Campos S.X., Bombardelli R.A., Cestari M.M. (2018). Exposure of male tilapia (*Oreochromis niloticus*) to copper by intraperitoneal injection: DNA damage and larval impairment. Aquat. Toxicol..

[30] Łuszczek-Trojnar E., Drag-Kozak E., Szczerbik P., Socha M., Popek W. (2014). Effect of long-term dietary lead exposure on some maturation and reproductive parameters of a female Prussian carp (*Carassius gibelio* B.). Environ. Sci. Pollut. Res..

[31] Johnson A., Carew E., Sloman K.A. (2007). The effects of copper on the morphological and functional development of zebrafish embryos. Aquat. Toxicol..

[32] Cao L., Huang W., Shan X., Xiao Z., Wang Q., Dou S. (2009). Cadmium toxicity to embryonic–larval development and survival in red sea bream *Pagrus major*. Ecotoxicol. Environ. Saf..

[33] Huang W., Cao L., Shan X., Xiao Z., Wang Q., Dou S. (2010). Toxic effects of zinc on the development, growth, and survival of red sea bream *Pagrus major* embryos and larvae. Arch. Environ. Contam. Toxicol..

[34] Zhang H., Cao H., Meng Y., Jin G., Zhu M. (2012). The toxicity of cadmium (Cd2+) towards embryos and pro-larva of soldatov’s catfish (*Silurus soldatovi*). Ecotoxicol. Environ. Saf..

[35] Witeska M., Sarnowski P., Ługowska K., Kowal E. (2014). The effects of cadmium and copper on embryonic and larval development of ide *Leuciscus idus* L. Fish Physiol. Biochem..

[36] Jezierska B., Ługowska K., Witeska M. (2009). The effects of heavy metals on embryonic development of fish (a review). Fish Physiol. Biochem..

[37] Rahman M.S., Islam S.M.M., Haque A., Shahjahan M. (2020). Toxicity of the organophosphate insecticide sumithion to embryo and larvae of zebrafish. Toxicol. Reports..

[38] Samson J.C., Shenker J. (2000). The teratogenic effects of methylmercury on early development of the zebrafish, *Danio rerio*. Aquat. Toxicol..

[39] Ashaf-Ud-Doulah M., Islam S.M.M., Zahangir M.M., Islam M.S., Brown C., Shahjahan M. (2021). Increased water temperature interrupts embryonic and larval development of Indian major carp rohu *Labeo rohita*. Aquac. Int..

[40] Nguyen L.T.H., Janssen C.R. (2002). Embryo-larval toxicity tests with the African catfish (*Clarias gariepinus*): comparative sensitivity of endpoints. Arch. Environ. Contam. Toxicol..

[41] Gárriz Á., Miranda L.A. (2020). Effects of metals on sperm quality, fertilization and hatching rates, and embryo and larval survival of pejerrey fish (*Odontesthes bonariensis*). Ecotoxicology..

[42] Osman A.G.M., Wuertz S., Mekkawy I.A., Exner H.-J., Kirschbaum F. (2007). Lead induced malformations in embryos of the African catfish *Clarias gariepinus* (Burchell, 1822). Environ. Toxicol..

[43] Hammerschmidt C.R., Sandheinrich M.B., Wiener J.G., Rada R.G. (2002). Effects of dietary methylmercury on reproduction of fathead minnows. Environ. Sci. Technol..

[44] Kong X., Jiang H., Wang S., Wu X., Fei W., Li L., Nie G., Li X. (2013). Effects of copper exposure on the hatching status and antioxidant defense at different developmental stages of embryos and larvae of goldfish *Carassius auratus*. Chemosphere..

[45] Mhadhbi L., Boumaiza M., Beiras R. (2010). A standard ecotoxicological bioassay using early life stages of the marine fish *Psetta maxima*. Aquat. Living Resour..

[46] Sassi A., Annabi A., Kessabi K., Kerkeni A., Saïd K., Messaoudi I. (2010). Influence of high temperature on cadmium-induced skeletal deformities in juvenile mosquitofish (*Gambusia affinis*). Fish Physiol. Biochem..

[47] Barjhoux I., Baudrimont M., Morin B., Landi L., Gonzalez P., Cachot J. (2012). Effects of copper and cadmium spiked-sediments on embryonic development of Japanese medaka (*Oryzias latipes*). Ecotoxicol. Environ. Saf..

[48] Kazlauskiene N., Vosyliene M.Z. (2008). Characteristic features of the effect of Cu and Zn mixtures on rainbow trout *Oncorhynchus mykiss*in ontogenesis. Polish J. Environ. Stud.

[49] El-Greisy Z.A., El-Gamal A.H.A. (2015). Experimental studies on the effect of cadmium chloride, zinc acetate, their mixture and the mitigation with vitamin C supplementation on hatchability, size and quality of newly hatched larvae of common carp, *Cyprinus carpio*. Egypt. J. Aquat. Res..

[50] Benaduce A.P.S., Kochhann D., Flores É.M.M., Dressler V.L., Baldisserotto B. (2008). Toxicity of cadmium for silver catfish *Rhamdia quelen* (Heptapteridae) embryos and larvae at different alkalinities. Arch. Environ. Contam. Toxicol..

[51] Gouva E., Nathanailides C., Skoufos I., Paschos I., Athanassopoulou F., Pappas I.S. (2020). Comparative study of the effects of heavy metals on embryonic development of zebrafish. Aquac. Res..

[52] Jurgelėnė Ž., Stankevičiūtė M., Kazlauskienė N., Baršienė J., Jokšas K., Markuckas A. (2019). Toxicological potential of cadmium impact on rainbow trout (*Oncorhynchus mykiss*) in early development. Bull. Environ. Contam. Toxicol..

[53] Green A.J., Mattingly C.J., Planchart A. (2017). Cadmium disrupts vestibular function by interfering with otolith formation. BioRxiv.

[54] Lizardo-Daudt H.M., Kennedy C. (2008). Effects of cadmium chloride on the development of rainbow trout *Oncorhynchus mykiss* early life stages. J. Fish Biol.

[55] Fraysse B., Mons R., Garric J. (2006). Development of a zebrafish 4-day embryo-larval bioassay to assess toxicity of chemicals. Ecotoxicol. Environ. Saf..

[56] Sarnowski P. (2003). The effect of metals on yolk sac resorption and growth of starved and fed common carp [*Cyprinus carpio* L.] larvae. Acta Sci. Pol. Piscaria..

[57] Metin C. (2001). Effects of aqueous cadmium on embryos and larvae of mirror carp. Indian J. Anim. Sci..

[58] Williams N.D., Holdway D.A. (2000). The effects of pulse-exposed cadmium and zinc on embryo hatchability, larval development, and survival of Australian crimson spotted rainbow fish (*Melanotaenia fluviatilis*). Environ. Toxicol..

[59] Mochida K., Ito K., Harino H., Onduka T., Kakuno A., Fujii K. (2008). Early life-stage toxicity test for copper pyrithione and induction of skeletal anomaly in a teleost, the mummichog (*Fundulus heteroclitus*). Environ. Toxicol. Chem..

[60] Ługowska K. (2007). The effects of copper and cadmium on embryonic development, and quality of newly hatched common carp (*Cyprinus carpio* L.) larvae. Electron. J. Polish Agric. Univ..

[61] Witeska M., Lugowska K. (2004). The effect of copper exposure during embryonic development on deformations of newly hatched common carp larvae, and further consequences. Electron. J. Polish Agric. Univ. Ser. Fish..

[62] Jezierska B., Lugowska K., Witeska M., Sarnowski P. (2000). Malformations of newly hatched common carp larvae. Electron. J. Polish Agric. Univ..

[63] Jahan N., Islam S.M.M., Rohani M.F., Hossain M.T., Shahjahan M. (2021). Probiotic yeast enhances growth performance of rohu (*Labeo rohita*) through upgrading hematology, and intestinal microbiota and morphology. Aquaculture..

[64] Islam S.M.M., Rohani M.F., Shahjahan M. (2021). Probiotic yeast enhances growth performance of Nile tilapia (*Oreochromis niloticus*) through morphological modifications of intestine. Aquac. Reports..

[65] Rohani M.F., Islam S.M., Hossain M.K., Ferdous Z., Siddik M.A., Nuruzzaman M., Padeniya U., Brown C., Shahjahan M. (2022). Probiotics, prebiotics and synbiotics improved the functionality of aquafeed: Upgrading growth, reproduction, immunity and disease resistance in fish. Fish Shellfish Immunol.

[66] Kucukbay Z., Yazlak H., Şahin N., Tuzcu M., Cakmak M., Gurdogan F., Juturu V., Sahin K. (2006). Zinc picolinate decreases oxidative stress in rainbow trout *Oncorhynchus mykiss*. Aquaculture..

[67] Liu X.J., Luo Z., Xiong B.X., Liu X., Zhao Y.H., Hu G.F., Lv G.J. (2010). Effect of waterborne copper exposure on growth, hepatic enzymatic activities and histology in *Synechogobius hasta*. Ecotoxicol. Environ. Saf..

[68] Asad F., Mubarik M.S., Ali T., Zahoor M.K., Ashrad R., Qamer S. (2019). Effect of organic and in-organic chromium supplementation on growth performance and genotoxicity of *Labeo rohita*. Saudi J. Biol. Sci..

[69] Aslam S., Yousafzai A.M. (2017). Chromium toxicity in fish: a review article, ~ 1483 ~. J. Entomol. Zool. Stud..

[70] Zha L.Y., Wang M.Q., Xu Z.R., Gu L.Y. (2007). Efficacy of chromium(III) supplementation on growth, body composition, serum parameters, and tissue chromium in rats. Biol. Trace Elem. Res..

[71] Wang M.Q., Xu Z.R., Zha L.Y., Lindemann M.D. (2007). Effects of chromium nanocomposite supplementation on blood metabolites, endocrine parameters and immune traits in finishing pigs. Anim. Feed Sci. Technol..

[72] Króliczewska B., Zawadzki W., Skiba T., Miśta D. (2005). Effects of chromium supplementation on chicken broiler growth and carcass characteristics. Acta Vet. Brno..

[73] Patil A., Palod J., Singh V.S., Kumar A. (2008). Effect of graded levels of chromium supplementation on certain serum biochemical parameters in broilers. Indian J. Anim. Sci..

[74] Ren M., Mokrani A., Liang H., Ji K., Xie J., Ge X., Liu B. (2018). Dietary chromium picolinate supplementation affects growth, whole-body composition, and gene expression related to glucose metabolism and lipogenesis in juvenile blunt snout bream, *Megalobrama amblycephala*. Biol. Trace Elem. Res..

[75] Yu-hua L. (2003). Effect of chromium on growth and plasma biochemical indexes of *Cyprinus carpio* juveniles. J. Dalian Fish. Univ..

[76] Selcuk Z., Tiril S.U., Alagil F., Belen V., Salman M., Cenesiz S., Muglali O.H., Yagci F.B. (2010). Effects of dietary l-carnitine and chromium picolinate supplementations on performance and some serum parameters in rainbow trout (*Oncorhynchus mykiss*). Aquac. Int..

[77] E.H. El-Sayed, E.I. Hassanein, M.H. Soliman, N.R. El-Khatib, The effect of dietary chromium picolinate on growth performance, blood parameters and immune status in Nile tilapia, *Oreochromis niloticus*, in: Proc. 3rd Glob. Fish. Aquac. Res. Conf. Foreign Agric. Relations (FAR), Egypt, 29 Novemb. - 1 December 2010, Massive Conferences and Trade Fairs, Cairo, 2010: pp. 51–63.

[78] Maret W., Krezel A. (2007). Cellular zinc and redox buffering capacity of metallothionein/thionein in health and disease. Mol. Med..

[79] Zhang Y.N., Wang S., Li K.C., Ruan D., Chen W., Xia W.G., Wang S.L., Abouelezz K.F.M., Zheng C.T. (2020). Estimation of dietary zinc requirement for laying duck breeders: effects on productive and reproductive performance, egg quality, tibial characteristics, plasma biochemical and antioxidant indices, and zinc deposition. Poult. Sci..

[80] Zhang T.Y., Liu J.L., Zhang J.L., Zhang N., Yang X., Qu H.X., Xi L., Han J.C. (2018). Effects of dietary zinc levels on the growth performance, organ zinc content, and zinc retention in broiler chickens. Rev. Bras. Ciência Avícola..

[81] Livingstone C. (2015). Zinc: physiology, deficiency, and parenteral nutrition.. Nutr. Clin. Pract. Off. Publ. Am. Soc. Parenter. Enter. Nutr..

[82] Eckerich C., Fackelmayer F.O., Knippers R. (2001). Zinc affects the conformation of nucleoprotein filaments formed by replication protein A (RPA) and long natural DNA molecules. Biochim. Biophys. Acta..

[83] Salim H.M., Lee H.R., Jo C., Lee S.K., Lee B.D. (2012). Effect of dietary zinc proteinate supplementation on growth performance, and skin and meat quality of male and female broiler chicks. Br. Poult. Sci..

[84] Yu H.R., Li L.Y., Shan L.L., Gao J., Ma C.Y., Li X. (2021). Effect of supplemental dietary zinc on the growth, body composition and anti-oxidant enzymes of coho salmon (*Oncorhynchus kisutch*) alevins. Aquac. Reports.

[85] Trevisan R., Flesch S., Mattos J.J., Milani M.R., Bainy A.C.D., Dafre A.L. (2014). Zinc causes acute impairment of glutathione metabolism followed by coordinated antioxidant defenses amplification in gills of brown mussels *Perna perna*. Comp. Biochem. Physiol. Part C Toxicol. Pharmacol..

[86] Huang F., Jiang M., Wen H., Wu F., Liu W., Tian J., Yang C. (2015). Dietary zinc requirement of adult Nile tilapia (*Oreochromis niloticus*) fed semi-purified diets, and effects on tissue mineral composition and antioxidant responses. Aquaculture..

[87] Liang J.-J., Yang H.-J., Liu Y.-J., Tian L.-X., Liang G.-Y. (2012). Dietary zinc requirement of juvenile grass carp (*Ctenopharyngodon idella*) based on growth and mineralization. Aquac. Nutr.

[88] Rider S.A., Davies S.J., Jha A.N., Clough R., Sweetman J.W. (2010). Bioavailability of co-supplemented organic and inorganic zinc and selenium sources in a white fishmeal-based rainbow trout (*Oncorhynchus mykiss*) diet. J. Anim. Physiol. Anim. Nutr. (Berl).

[89] Vielma J., Ruohonen K., Peisker M. (2002). Dephytinization of two soy proteins increases phosphorus and protein utilization by rainbow trout, *Oncorhynchus mykiss*. Aquaculture..

[90] Shiau S.Y., Jiang L.C. (2006). Dietary zinc requirements of grass shrimp, *Penaeus monodon*, and effects on immune responses. Aquaculture..

[91] Xiong D., Fang T., Yu L., Sima X., Zhu W. (2011). Effects of nano-scale TiO2, ZnO and their bulk counterparts on zebrafish: Acute toxicity, oxidative stress and oxidative damage. Sci. Total Environ..

[92] Do Carmo M.V., Sá E., Pezzato L.E., Ferreira Lima M.M.B., De Magalhães Padilha P. (2004). Optimum zinc supplementation level in Nile tilapia *Oreochromis niloticus* juveniles diets. Aquaculture..

[93] Tan L.-N., Feng L., Liu Y., Jiang J., Jiang W.-D., Hu K., Li S.-H., Zhou X.-Q. (2011). Growth, body composition and intestinal enzyme activities of juvenile Jian carp (*Cyprinus carpio var.* Jian) fed graded levels of dietary zinc. Aquac. Nutr.

[94] Luo Z., Tan X.Y., Zheng J.L., Chen Q.L., Liu C.X. (2011). Quantitative dietary zinc requirement of juvenile yellow catfish *Pelteobagrus fulvidraco*, and effects on hepatic intermediary metabolism and antioxidant responses. Aquaculture..

[95] Salvaggio A., Marino F., Albano M., Pecoraro R., Camiolo G., Tibullo D., Bramanti V., Lombardo B.M., Saccone S., Mazzei V., Brundo M.V. (2016). Toxic effects of zinc chloride on the bone development in *Danio rerio* (Hamilton, 1822). Front. Physiol..

[96] Mohseni M., Pourkazemi M., Bai S.C. (2014). Effects of dietary inorganic copper on growth performance and immune responses of juvenile beluga, *Huso huso*. Aquac. Nutr..

[97] El Basuini M.F., El-Hais A.M., Dawood M.A.O., Abou-Zeid A.E.S., EL-Damrawy S.Z., Khalafalla M.M.E.S., Koshio S., Ishikawa M., Dossou S. (2016). Effect of different levels of dietary copper nanoparticles and copper sulfate on growth performance, blood biochemical profiles, antioxidant status and immune response of red sea bream (*Pagrus major*). Aquaculture..

[98] Sabatini S.E., Chaufan G., Juárez Á.B., Coalova I., Bianchi L., Eppis M.R., del M., Ríos de Molina C. (2009). Dietary copper effects in the estuarine crab, *Neohelice granulata* (Chasmagnathus), maintained at two different salinities. Comp. Biochem. Physiol. Part C Toxicol. Pharmacol..

[99] Lin Y.H., Shie Y.Y., Shiau S.Y. (2008). Dietary copper requirements of juvenile grouper,*Epinephelus malabaricus*. Aquaculture..

[100] Wang L., Wang J., Bharadwaj A.S., Xue M., Qin Y., Wu X., Zheng Y., Han F. (2015). Effects of dietary copper sources on growth, tissue copper accumulation and physiological responses of Japanese sea bass (*Lateolabrax japonicus*) (Cuvier, 1828) fed semipurified or practical diets. Aquac. Res..

[101] Wang J., Ai Q., Mai K., Xu H., Zuo R. (2014). Dietary chromium polynicotinate enhanced growth performance, feed utilization, and resistance to *Cryptocaryon irritans* in juvenile large yellow croaker (*Larmichthys crocea*). Aquaculture..

[102] Lanno R.P., Slinger S.J., Hilton J.W. (1985). Maximum tolerable and toxicity levels of dietary copper in rainbow trout (*Salmo gairdneri* Richardson). Aquaculture..

[103] Shukla V., Dhankhar M., Prakash J., Sastry K.V. (2007). Bioaccumulation of Zn, Cu and Cd in *Channa punctatus*. J. Environ. Biol.

[104] Crichton R.R. (1991).

[105] Erickson R.J., Mount D.R., Highland T.L., Hockett J.R., Leonard E.N., Mattson V.R., Dawson T.D., Lott K.G. (2010). Effects of copper, cadmium, lead, and arsenic in a live diet on juvenile fish growth. Can. J. Fish. Aquat. Sci..

[106] Fazio F., Habib S.S., Naz S., Hashmi M.A.H., Saoca C., Ullah M. (2021). Cadmium sub-lethal concentration effect on growth, haematological and biochemical parameters of *Mystus seenghala* (Sykes, 1839). Biol. Trace Elem. Res..

[107] Paul J.S., Small B.C. (2021). Chronic exposure to environmental cadmium affects growth and survival, cellular stress, and glucose metabolism in juvenile channel catfish (*Ictalurus punctatus*). Aquat. Toxicol..

[108] Xie D., Li Y., Liu Z., Chen Q. (2019). Inhibitory effect of cadmium exposure on digestive activity, antioxidant capacity and immune defense in the intestine of yellow catfish (*Pelteobagrus fulvidraco*). Comp. Biochem. Physiol. Part C Toxicol. Pharmacol..

[109] Ayyat M.S., Mahmoud H.K., El-Hais A.E.A.M., Abd El-Latif K.M. (2017). The role of some feed additives in fish fed on diets contaminated with cadmium. Environ. Sci. Pollut. Res..

[110] Yuan S.S., Lv Z.M., Zhu A.Y., Zheng J.L., Wu C.W. (2017). Negative effect of chronic cadmium exposure on growth, histology, ultrastructure, antioxidant and innate immune responses in the liver of zebrafish: preventive role of blue light emitting diodes. Ecotoxicol. Environ. Saf..

[111] Zheng J.L., Yuan S.S., Wu C.W., Li W.Y. (2016). Chronic waterborne zinc and cadmium exposures induced different responses towards oxidative stress in the liver of zebrafish. Aquat. Toxicol..

[112] Abdel-Tawwab M., Wafeek M. (2014). Influence of water temperature and waterborne cadmium toxicity on growth performance and metallothionein–cadmium distribution in different organs of Nile tilapia, *Oreochromis niloticus* (L.). J. Therm. Biol..

[113] Heydarnejad M.S., Khosravian-Hemamai M., Nematollahi A. (2013). Effects of cadmium at sub-lethal concentration on growth and biochemical parameters in rainbow trout (*Oncorhynchus mykiss*). Ir. Vet. J.

[114] Ahmed M.S., Ahmed K.S., Mehmood R., Ali H., Khan W.A. (2012). Low dose effects of cadmium and lead on growth in fingerlings of a vegetarian fish, grass carp (*ctenopharyngodon idella*). J. Anim. Plant Sci.

[115] Tan X.Y., Luo Z., Zhang G.Y., Liu X.J., Jiang M. (2010). Effect of dietary cadmium level on the growth, body composition and several hepatic enzymatic activities of juvenile yellow catfish, *Pelteobagrus fulvidraco*. Aquac. Res..

[116] Giri A.K., Sahu N.P., Dash G. (2021). Improvement in the growth status and carbohydrate utilization of *Labeo rohita* (Hamilton, 1822) fingerlings with dietary supplementation of chromium picolinate. Fish Physiol. Biochem..

[117] Mohamed A.A.R., El-Houseiny W., EL-Murr A.E., Ebraheim L.L.M., Ahmed A.I., El-Hakim Y.M.A. (2020). Effect of hexavalent chromium exposure on the liver and kidney tissues related to the expression of CYP450 and GST genes of *Oreochromis niloticus* fish: role of curcumin supplemented diet. Ecotoxicol. Environ. Saf..

[118] Ko H.D., Park H.J., Kang J.C. (2019). Change of growth performance, hematological parameters, and plasma component by hexavalent chromium exposure in starry flounder, *Platichthys stellatus*. Fish. Aquat. Sci..

[119] Ren M., Mokrani A., Liang H., Ji K., Xie J., Ge X., Liu B. (2018). Dietary chromium picolinate supplementation affects growth, whole-body composition, and gene expression related to glucose metabolism and lipogenesis in juvenile blunt snout bream, *Megalobrama amblycephala*. Biol. Trace Elem. Res..

[120] Kim J.H., Kang J.C. (2016). The chromium accumulation and its physiological effects in juvenile rockfish, *Sebastes schlegelii*, exposed to different levels of dietary chromium (Cr6+) concentrations. Environ. Toxicol. Pharmacol..

[121] Ahmed A.R., Moody A.J., Fisher A., Davies S.J. (2013). Growth performance and starch utilization in common carp (*Cyprinus carpio* L.) in response to dietary chromium chloride supplementation. J. Trace Elem. Med. Biol..

[122] Mehrim A.I. (2012). Effect of dietary chromium picolinate supplementation on growth performance, carcass composition and organs indices of Nile tilapia (*Oreochromis niloticus* L.) fingerlings. J. Fish. Aquat. Sci..

[123] Liu T., Wen H., Jiang M., Yuan D., Gao P., Zhao Y., Wu F., Liu W. (2010). Effect of dietary chromium picolinate on growth performance and blood parameters in grass carp fingerling, *Ctenopharyngodon idellus*. Fish Physiol. Biochem..

[124] Mishra A.K., Mohanty B. (2008). Histopathological effects of hexavalent chromium in the ovary of a fresh water fish, *Channa punctatus* (Bloch). Bull. Environ. Contam. Toxicol..

[125] Ghosh A., Ali S., Mukherjee S.K., Saha S., Kaviraj A. (2020). Bioremediation of Copper and Nickel from Freshwater Fish *Cyprinus carpio* Using Rhiozoplane Bacteria Isolated from *Pistia stratiotes*. Environ. Process..

[126] Linag H., Ji K., Ge X., Mi H., Xi B., Ren M. (2020). Effects of dietary copper on growth, antioxidant capacity and immune responses of juvenile blunt snout bream (*Megalobrama amblycephala*) as evidenced by pathological examination. Aquac. Reports.

[127] Shokr E.A.M. (2020). Effect of copper on hematological, biochemical changes and reproductive hormones of the nile tilapia *oreochromis niloticus*. Egypt. J. Aquat. Biol. Fish..

[128] Ghasemzadeh A., Bahrekazemi M. (2019). The reduction of CuSO4 toxicity in common carp (*Cyprinus carpio* linnaeus, 1758) after pre-exposure to CaCO3. Asian Fish. Sci..

[129] Zebral Y.D., Anni I.S.A., Afonso S.B., Abril S.I.M., Klein R.D., Bianchini A. (2018). Effects of life-time exposure to waterborne copper on the somatotropic axis of the viviparous fish *Poecilia vivipara*. Chemosphere..

[130] Abdel-Hameid N.A.H., Zehra S., Khan M.A. (2017). Dietary copper requirement of fingerling *Channa punctatus* (Bloch) based on growth, feed conversion, blood parameters and whole body copper concentration. Aquac. Res..

[131] Sevcikova M., Modra H., Blahova J., Dobsikova R., Plhalova L., Zitka O., Hynek D., Kizek R., Skoric M., Svobodova Z. (2016). Biochemical, haematological and oxidative stress responses of common carp (*Cyprinus carpio* L.) after sub-chronic exposure to copper. Vet. Med. (Praha)..

[132] Jiang H., Kong X., Wang S., Guo H. (2016). Effect of copper on growth, digestive and antioxidant enzyme activities of juvenile qihe crucian carp, carassius carassius, during exposure and recovery. Bull. Environ. Contam. Toxicol..

[133] Moosavi M.J., Shamushaki V.-A.J. (2015). Effects of different levels of copper sulfate on growth and reproductive performances in guppy (*P. reticulate*). J. Aquac. Res. Dev..

[134] Tang Q.Q., Feng L., Jiang W.D., Liu Y., Jiang J., Li S.H., Kuang S.Y., Tang L., Zhou X.Q. (2013). Effects of dietary copper on growth, digestive, and brush border enzyme activities and antioxidant defense of hepatopancreas and intestine for young grass carp (*Ctenopharyngodon idella*). Biol. Trace Elem. Res..

[135] Shao X.P., Bin Liu W., Le K., Xu Lu, W.N., Zhang W.W., Wang Y., Zhu J. (2012). Effects of tribasic copper chloride on growth, copper status, antioxidant activities, immune responses and intestinal microflora of blunt snout bream (*Megalobrama amblycephala*) fed practical diets. Aquaculture.

[136] Kim S.G., Kang J.C. (2004). Effect of dietary copper exposure on accumulation, growth and hematological parameters of the juvenile rockfish, *Sebastes schlegeli*. Mar. Environ. Res..

[137] Hansen J.A., Welsh P.G., Lipton J., Suedkamp M.J. (2002). The effects of long-term cadmium exposure on the growth and survival of juvenile bull trout (*Salvelinus confluentus*). Aquat. Toxicol..

[138] Uzo-God O.C., Agarwal A., Singh N.B. (2019). Effects of dietary nano and macro iron oxide (Fe2O3) on the growth, biochemical, and hematological profiles of African catfish (*Clarias gariepinus*) fingerlings. J. Appl. Aquac..

[139] Guo Y.L., Jiang W.D., Wu P., Liu Y., Zhou X.Q., Kuang S.Y., Tang L., Tang W.N., Zhang Y.A., Feng L. (2017). The decreased growth performance and impaired immune function and structural integrity by dietary iron deficiency or excess are associated with TOR, NF-κB, p38MAPK, Nrf2 and MLCK signaling in head kidney, spleen and skin of grass carp. Fish Shellfish Immunol.

[140] Ling J., Feng L., Liu Y., Jiang J., Jiang W.-D., Hu K., Li S.-H., Zhou X.-Q. (2010). Effect of dietary iron levels on growth, body composition and intestinal enzyme activities of juvenile Jian carp (*Cyprinus carpio* var. Jian). Aquac. Nutr.

[141] Ye C.-X., Liu Y.-J., Mai K.-S., Tian L.-X., Yang H.-J., Niu J., Huang J.-W. (2007). Effect of dietary iron supplement on growth, haematology and microelements of juvenile grouper, *Epinephelus coioides*. Aquac. Nutr..

[142] Barros M.M., Lim C., Evans J.J., Klesius P.H. (2000). Effect of iron supplementation to cottonseed meal diets on the growth performance of channel catfish, *Ictalurus punctatus*. J. Appl. Aquac..

[143] Lim C., Klesius P.H., Li M.H., Robinson E.H. (2000). Interaction between dietary levels of iron and vitamin C on growth, hematology, immune response and resistance of channel catfish (*Ictalurus punctatus*) to *Edwardsiella ictaluri* challenge. Aquaculture..

[144] Maage A., Julshamn K., Berge G.E. (2001). Zinc gluconate and zinc sulphate as dietary zinc sources for Atlantic salmon. Aquac. Nutr.

[145] Zulfahmi I., Rahmi A., Muliari M., Akmal Y., Paujiah E., Sumon K.A., Rahman M.M. (2021). Exposure to lead nitrate alters growth and haematological parameters of milkfish (*Chanos chanos*). Bull. Environ. Contam. Toxicol..

[146] Javed M. (2012). Effects of zinc and lead toxicity on the growth and their bioaccumulation in fish. Pak. Vet. J..

[147] Ando H., Ogawa S., Shahjahan M., Ikegami T., Doi H., Hattori A., Parhar I. (2014). Diurnal and circadian oscillations in expression of kisspeptin, kisspeptin receptor and gonadotrophin-releasing hormone 2 genes in the grass puffer, a semilunar-synchronised spawner. J. Neuroendocrinol..

[148] Ando H., Shahjahan M., Hattori A. (2013). Molecular neuroendocrine basis of lunar-related spawning in grass puffer. Gen. Comp. Endocrinol..

[149] Ando H., Shahjahan M., Kitahashi T. (2018). Periodic regulation of expression of genes for kisspeptin, gonadotropin-inhibitory hormone and their receptors in the grass puffer: implications in seasonal, daily and lunar rhythms of reproduction. Gen. Comp. Endocrinol..

[150] Li Z.H., Li P., Dzyuba B., Randak T. (2010). Influence of environmental related concentrations of heavy metals on motility parameters and antioxidant responses in sturgeon sperm. Chem. Biol. Interact..

[151] Dietrich G.J., Ciereszko A., Kowalski R.K., Rzemieniecki A., Bogdan E., Demianowicz W., Dietrich M., Kujawa R., Glogowski J. (2012). Motility and fertilizing capacity of frozen/thawed sperm of Siberian sturgeon after a short-time exposure of fresh semen to mercury and cadmium. J. Appl. Ichthyol..

[152] Dietrich G.J., Dietrich M., Kowalski R.K., Dobosz S., Karol H., Demianowicz W., Glogowski J. (2010). Exposure of rainbow trout milt to mercury and cadmium alters sperm motility parameters and reproductive success. Aquat. Toxicol..

[153] Hayati A., Giarti K., Winarsih Y., Amin M.H.F. (2017). The effect ef cadmium on sperm quality and fertilization of *Cyprinus carpio* L. J. Trop. Biodivers. Biotechnol..

[154] Velma V., Vutukuru S.S., Tchounwou P.B. (2009). Ecotoxicology of hexavalent chromium in freshwater fish: a critical review. Rev. Environ. Health.

[155] Ni X., Shen Y. (2021). Transgenerational effects of hexavalent chromium on marine medaka (*Oryzias melastigma*) reveal complex transgenerational adaptation in offspring. Biomolecules..

[156] Chen H., Cao J., Li L., Wu X., Bi R., Klerks P.L., Xie L. (2016). Maternal transfer and reproductive effects of Cr(VI) in Japanese medaka (*Oryzias latipes*) under acute and chronic exposures. Aquat. Toxicol..

[157] Liao C.Y., Fu J.J., Shi J.B., Zhou Q.F., Yuan C.G., Bin Jiang G. (2006). Methylmercury accumulation, histopathology effects, and cholinesterase activity alterations in medaka (*Oryzias latipes*) following sublethal exposure to methylmercury chloride. Environ. Toxicol. Pharmacol..

[158] Abascal F.J., Cosson J., Fauvel C. (2007). Characterization of sperm motility in sea bass: the effect of heavy metals and physicochemical variables on sperm motility. J. Fish Biol..

[159] Alsop D., Brown S., Van Der Kraak G. (2007). The effects of copper and benzo[a]pyrene on retinoids and reproduction in zebrafish. Aquat. Toxicol..

[160] James R., Sampath K., Jothilakshmi S., Vasudhevan I., Thangarathinam R. (2008). Effects of copper toxicity on growth, reproduction and metal accumulation in chosen ornamental fishes. Ecohydrol. Hydrobiol..

[161] Hani Y.M.I., Turies C., Palluel O., Delahaut L., Bado-Nilles A., Geffard A., Dedourge-Geffard O., Porcher J.M. (2019). Effects of a chronic exposure to different water temperatures and/or to an environmental cadmium concentration on the reproduction of the threespine stickleback (*Gasterosteus aculeatus*). Ecotoxicol. Environ. Saf..

[162] Drevnick P.E., Sandheinrich M.B. (2003). Effects of dietary methylmercury on reproductive endocrinology of fathead minnows. Environ. Sci. Technol..

[163] Van Look K.J.W., Kime D.E. (2003). Automated sperm morphology analysis in fishes: the effect of mercury on goldfish sperm. J. Fish Biol.

[164] Arnold B.S. (2000).

[165] Chyb J., Mikolajczyk T., Szczerbik P., Epler P. (2000). The influence of zinc on sperm motility of common carp - a computer assisted studies. Arch. Rybactwa Pol..

